# Recursive neural programs: A differentiable framework for learning compositional part-whole hierarchies and image grammars

**DOI:** 10.1093/pnasnexus/pgad337

**Published:** 2023-10-14

**Authors:** Ares Fisher, Rajesh P N Rao

**Affiliations:** Paul G. Allen School of Computer Science and Engineering, University of Washington, Seattle, WA 98195, USA; Paul G. Allen School of Computer Science and Engineering, University of Washington, Seattle, WA 98195, USA

**Keywords:** artificial intelligence, program synthesis, deep learning, cognitive science

## Abstract

Human vision, thought, and planning involve parsing and representing objects and scenes using structured representations based on part-whole hierarchies. Computer vision and machine learning researchers have recently sought to emulate this capability using neural networks, but a generative model formulation has been lacking. Generative models that leverage compositionality, recursion, and part-whole hierarchies are thought to underlie human concept learning and the ability to construct and represent flexible mental concepts. We introduce Recursive Neural Programs (RNPs), a neural generative model that addresses the part-whole hierarchy learning problem by modeling images as hierarchical trees of probabilistic sensory-motor programs. These programs recursively reuse learned sensory-motor primitives to model an image within different spatial reference frames, enabling hierarchical composition of objects from parts and implementing a grammar for images. We show that RNPs can learn part-whole hierarchies for a variety of image datasets, allowing rich compositionality and intuitive parts-based explanations of objects. Our model also suggests a cognitive framework for understanding how human brains can potentially learn and represent concepts in terms of recursively defined primitives and their relations with each other.

**Significance Statement**

A crucial aspect of intelligent, symbolic behavior is the ability to recursively compose known elements into unseen objects and representations, enabling imagination, language, and other creative abilities. A primary limitation of modern deep learning models is the lack of such explicit compositionality, which also makes their learned representations very difficult to interpret. We introduce a novel model architecture that learns to represent images as recursive transformations of differentiable “programs,” allowing interpretable and intuitive generation of images through a process resembling a visual grammar.

## Introduction

Human visual cognition exploits hierarchical relationships between objects and their parts. For example, a human face can be modeled as a hierarchical tree of parts, each part’s relative position specified within a local reference frame: eyes, nose, mouth, etc. positioned within the face’s reference frame, the parts of an eye (such as eyebrow, eyelid, iris, and pupil) positioned within the eye’s reference frame, and so on. To emulate such a capability, a computer vision system needs to not only learn what a part looks like (as in current deep convolutional networks) but also the relative transformations of the parts within a local reference frame, and do this recursively in order to compose a human face (or a Picasso painting).

**Fig. 1.**
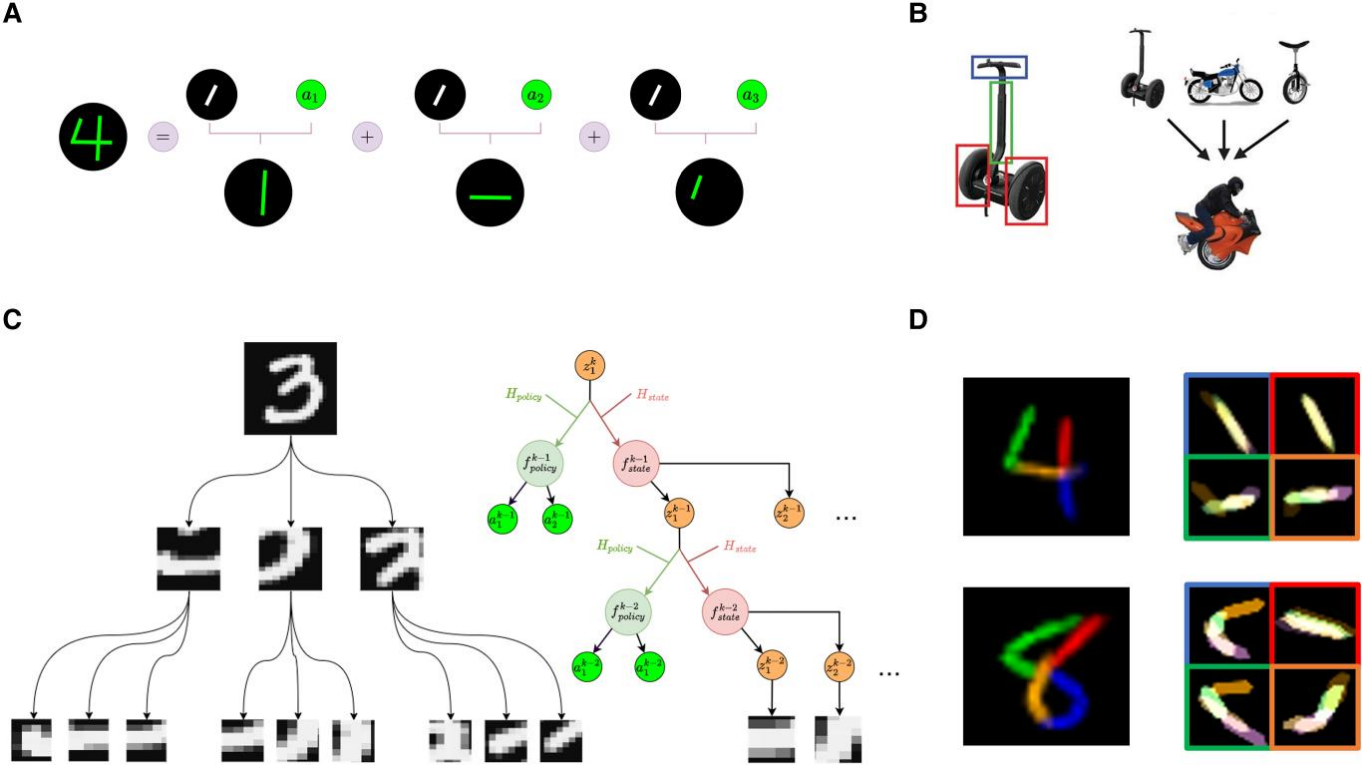
Parsing and generating images with a sequence of transformed primitives. A) A “4” can be constructed by generating three identical straight lines (within black circles) and transforming them according to parameters a to place them in the appropriate locations within a reference frame for the digit. B) Humans can recognize individual parts of objects (left) and transform them in new ways to create novel objects (right). (Adapted from (1)). C) Left: A handwritten digit from the MNIST dataset decomposed into an abstraction tree of parts, each part being further decomposed into smaller subparts. Right: Schematic representation of the hierarchical structure of a recursive neural program. A higher-level “program,” represented here by the vector $z^{k}$ at level k, generates (via hypernetworks $H_{\mathrm{state}}$ and $H_{\mathrm{policy}}$) the lower-level state and action *functions*  $f_{state}^{k-1}$ and $f_{\mathrm{policy}}^{k-1}$ respectively, to construct an object (e.g. a digit) by generating a sequence of parts $z^{k-1}$ and transforming them according to their respective action vectors $a^{k-1}$ (transformation parameters such as position, scale, etc.). Each part $z^{k-1}$ is in turn a program vector which generates (via state and action networks for level k−2) the subparts $z^{k-2}$, transformed according to $a^{k-2}$, within that part’s reference frame. The recursive program reaches a chosen depth (here k−2), whereupon the latent vector $z^{k-2}$ is decoded into an image patch using a network parameterized by $z^{k-1}$. D) Two example images generated by a RNP showing how the model can learn to construct digits by sequential transformation of multiple copies of a single part (here, a straight line). Top: Demonstration that the idea expressed in (A) can be learned by RNPs: a “4” reconstructed by transforming four strokes, each of which is made up of four transformed lines (right panel, bordered boxes). Bottom: A three-level architecture (as above) can generate more complex digits like an “8”. For both digits, the strokes were generated in the order blue → red → green → orange.

Beyond vision, nested structure and hierarchical parts-based decompositions are ubiquitous in human attributes such as natural language (texts, chapters, paragraphs, sentences, words, characters) and complex behaviors (such as cooking a recipe or driving to work). For example, driving to work consists of “high-level” behaviors (e.g. get to the car, start the car, etc.), which are in turn composed of “lower-level” behaviors such as “walk to the house door,” “open the door,” etc. which are in turn composed of other actions: “put left foot in front of the right,” “grasp door handle,” and so on. Recursive modeling confers the important property of compositionality (1–3): the same building blocks can be hierarchically and recursively composed into an endless variety of possible patterns, allowing an agent to “imagine” novel configurations of parts (e.g. for creating new solutions to problems), and recognize new configurations of known parts for zero-shot generalization (Fig. 1A,B). The challenge lies in learning a model of the parts and their transformations that is recursive and composable. Generative models (4–9) capture rich structure to represent images but are not explicitly composable. Previous studies have used various approaches to exploit the compositionality of images, including bilinear sparse coding (10–14) and Lie groups (15–17). However, these models do not capture the recursive and tree-like structure of the modeled data. Existing approaches for parsing and generating tree-structured data such as images and natural language (1, 18–23) are either not recursive (18, 21), not compositional (8, 9, 18, 22), not generative (19, 20), or not differentiable (1, 23). Indeed, the lack of a differentiable “program space” has been a major challenge in the field.

Here, we introduce recursive neural programs (RNPs), which address these problems using a generative model for fully differentiable, recursive tree representations of objects and their parts. Our model relies on hypernetworks (24), which are neural networks that generate parameters for other neural networks, to generate neural programs. Our model also builds on recent work on Active Predictive Coding (25–27) in using a state and action hierarchy but is fully generative, recursive, and probabilistic, allowing a structured variational approach to inference and sampling of neural programs. The key differences between our approach and existing approaches are: (i) Our approach can be extended to arbitrary tree depth, creating a grammar for images that can be recursively applied (Fig. 1C), (ii) our approach provides a sensible way to perform gradient descent in hierarchical “program” space, and (iii) our model can be made adaptive by letting information flow from children to parents in the tree, e.g. via prediction errors, emulating predictive coding models (26–29).

The architecture of our model departs from conventional neural networks in that it generates sequences in a recursive and hierarchical fashion. A latent state at one level of our model generates a sequence of states at the level below, with each lower-level state itself representing a sequence. For example, in representing a human face, a latent state can correspond to an entire face, its sub-states could correspond to features (like eyes, nose, etc.), and their sub-states in turn can correspond to lines, curves, and other primitives that are composed to form these features. Each state consists of two attributes: a sequence of lower-level states (here, image primitives or features), and a sequence of “actions” which correspond to transformations of these states (e.g. rotating or translating a line or a nose on an image “canvas”). We call a function that generates state sequences a state transition function and the corresponding action generating function a “policy.” In our model, each state generates both a transition function and a policy, and therefore represents a “program” for generating states and actions. In a hierarchical, recursive setting, such programs allow for abstraction: for example, a program for drawing a nose does not need to “know” about the placement of the nose or a whole face, only the subparts that will result in a nose.

The separation between states and actions is characteristic of recent conceptual models of vision and action (20, 25, 26). It is also inspired by the observation that the cortical visual processing pathway is roughly separated into two streams: a “what” or ventral stream that flows into the inferior temporal cortex, and a “where” or dorsal stream that flows into the posterior parietal cortex (30). Although there exist interactions between the two streams, making the separation less clear-cut than originally believed, the separation does conceptually lend itself to recursion and compositionality: object features (“what”) can be used at any scale to compose a new object by transforming each feature (“where”) independently of other features, and a new scene can be created by recursively applying this idea to the newly composed objects. Our model makes testable predictions for the function of the cortical visual system: the model predicts cortical representations of objects are inherently sensory-motor, i.e. they explicitly contain information regarding sensory features as well as their transformations. This is consistent with recent theories about the cortex (27, 31). Our model also suggests the need to bind the information between the dorsal and ventral streams into state representations at multiple hierarchical levels, a prediction that is in line with recent suggestions regarding the role of the hippocampus in binding cortical “what” and “where” information (32).

## Recursive neural programs

To illustrate our approach, we describe a 3-level RNP, though the recursive nature of the architecture can easily be generalized to more levels (Algorithm 1). Consider the problem of parsing an image of a digit using three levels of an abstraction tree (Fig. 1C (left)), e.g. in terms of a full digit level (k=2), a parts level (k=1) and a subparts (or strokes) level (k=0). A top-level vector representation (at k=2) generates the digit using a lower-level neural program (at k=1) that generates parts and their transformations within the digit’s reference frame (31). The lower-level neural program is generated by the top-level representation using hypernetworks (see below). Each part in turn is generated by a further lower-level neural program (at k=0) that generates a sequence of subparts and their transformations within that part’s reference frame.

**Algorithm 1:** Recursive Neural Program

1: **procedure** RNP (input=x,levels=K)
2: μ,logvar=Encoder(x)
3: $z^{K}∼N(\mu ,\exp\;(logvar))$
4: **return** RNPdecoder($z^{K},K$)
5: **procedure** RNPdecoder(z,level=k)
6: $enc_{state}^{k-1},f_{state}^{k-1},dec_{state}^{k-1},z_{0}^{k-1}\leftarrow H_{state}(z)$
7: $enc_{policy}^{k-1},f_{policy}^{k-1},dec_{policy}^{k-1},a_{0}^{k-1}\leftarrow H_{policy}(z)$
8: $p_{t}^{k-1}=0$ ⊳ canvas for image reconstruction
9: **for** $t=1:\tau^{k-1}$ **do**
10: $z_{t}^{k-1}=f_{state}^{k-1}(a_{t-1}^{k-1},z_{t-1}^{k-1})$
11: $a_{t}^{k-1}=f_{policy}^{k-1}(a_{t-1}^{k-1},z_{t-1}^{k-1})$
12: ${\hat{x}}_{t}^{k-1}=dec_{state}^{k-1}(z_{t}^{k-1})$
13: **if** k>0 **then**
14: $p_{t}^{k-1}\leftarrow p_{t-1}^{k-1}+RNPdecoder(z_{t}^{k-1},k-1)$
15: **else**
16: $p_{t}^{k-1}\leftarrow p_{t-1}^{k-1}+g({\hat{x}}_{t}^{k-1},a_{t}^{k-1})$
17: **return** $p_{t}^{k-1}$

**Fig. 2.**
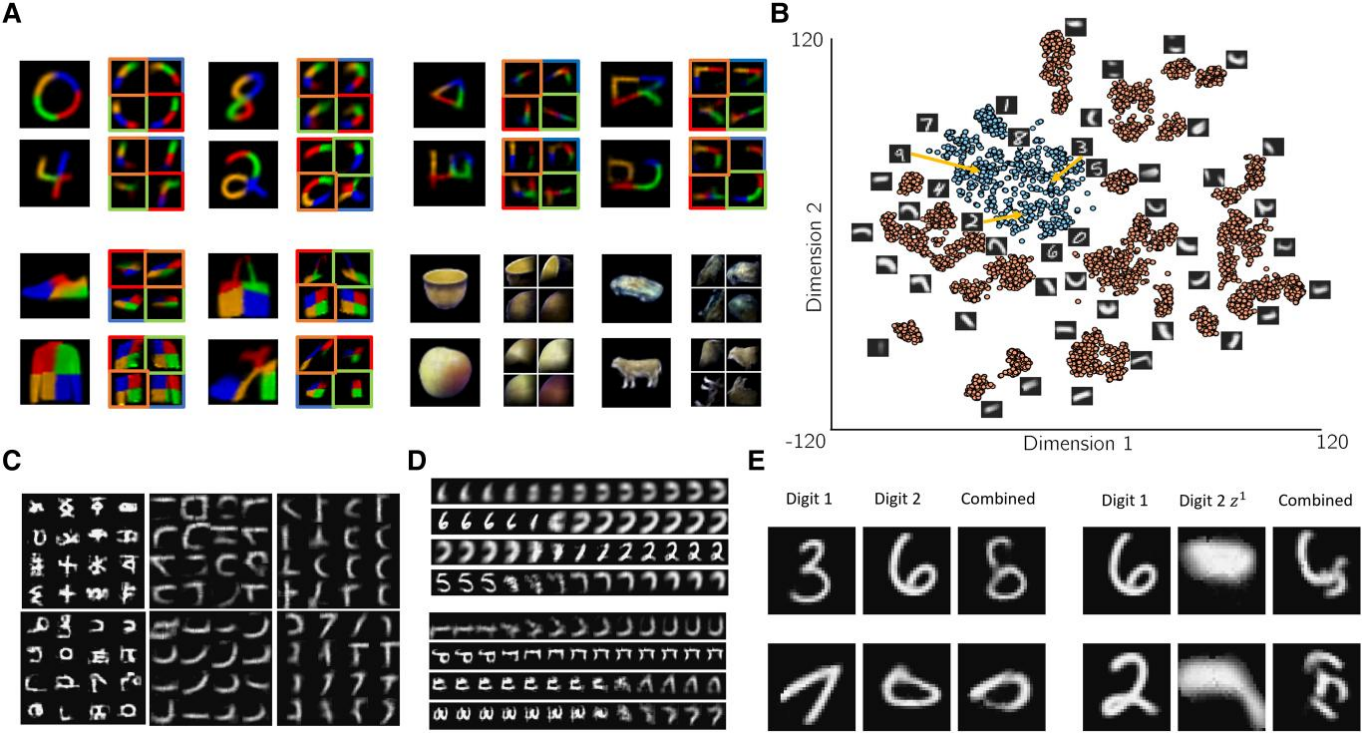
Hierarchical parts-based decomposition and clustering in neural program space A) Parsing of MNIST digits (top left), Omniglot characters (top right), Fashion-MNIST objects (bottom left), and ETH-80 objects (bottom right) by trained RNP models. Two levels of the hierarchical representation are shown: parts generated by each $z^{2}$ (left: each part is denoted by a different color); and subparts generated by each $z^{1}$ (right, bordered boxes, each subpart is denoted by a different color). Order as in Fig. 1D. Each bordered box shows the output of a program generated by $z^{1}$ to construct a part as a combination of subparts. B) t-SNE clustering of $z^{2}$ and $z^{1}$ vectors in an RNP model trained on MNIST. A representative image is shown for each cluster. Note that the $z^{2}$ vectors (blue) cluster separately from the $z^{1}$ vectors (orange). $z^{0}$ vectors, which also occupy the same space as $z^{2}$ and $z^{1}$ in the recursive model, are omitted to prevent clutter. C) Example clusters of sampled images from $z^{2}$ (leftmost column) and $z^{1}$ (remaining columns), sampled unconditionally from the generative model learned by an RNP trained on Omniglot, illustrating a variety of digit and part-level representations. D) Example linear interpolations in z space from the center of one cluster (leftmost image) to the center of another cluster (rightmost image), showing novel generated images from neural programs in the intermediate space. Left: MNIST; right: Omniglot. E) Left: Example images (third column) created by combinations of two $z^{2}$ vectors generated by an encoder (first two columns). Right: As in left, but first $z^{2}$ vector is combined with a $z^{1}$ vector from another digit.

The “neural program” at each level is implemented by two mutually interacting recurrent neural networks, one implementing a state transition function (or “forward model” for the state) that predicts the next state $z_{t+1}^{k}=f_{\mathrm{state}}^{k}(z_{t}^{k},a_{t}^{k})$; and another implementing an action function (“policy”) $a_{t+1}^{k}=f_{\mathrm{policy}}^{k}(z_{t}^{k},a_{t}^{k})$ (Fig. 1C (right), Fig. 2, Algorithm 1). In this article, we assume actions correspond to transformations of parts, but the framework is more general and can be applied to other problems as well (e.g. planning (27)). The state transition and policy functions in our model follow the framework used in a partially observable Markov decision process (POMDP) (33). At each time point, $f_{\mathrm{state}}^{k}$ and $f_{\mathrm{policy}}^{k}$ receive as input the state-action pair $\left(z_{t}^{k},a_{t}^{k}\right)$ from the previous timepoint. For image modeling, using both the state vector and action vector (affine transforms) as inputs helps disambiguate between identical patches that could be used in different locations: for example, a straight line (state) can occur in multiple locations (actions) in the digit “7”.

We use hypernetworks (24) to provide our framework with the flexibility to create different “programs” for different levels of abstraction. Hypernetworks are neural networks that generate the parameters for other neural networks, creating networks specialized for a specific task (encoded as a vector input to the hypernetwork). In the RNP model, we use two hypernetworks, a “state hypernetwork” $H_{\mathrm{state}}$ and a “policy hypernetwork” $H_{\mathrm{policy}}$, which are together responsible for generating the corresponding parts given a higher-level program (state vector) as input. Each hypernetwork generates a network whose components include an encoder, a recurrent network (RNN) and a decoder. At each time-step, the encoder receives the state-action pair generated in the previous time-step and feeds it to the RNN, which in turn generates the next state or action; Figs. 1 and 5). The decoder then decodes the state or action into an image patch or affine transform parameters, respectively. After each time-step, the resultant image patch is transformed by the affine transform parameters to be placed in the desired configuration within the state’s reference frame (“canvas”). For an MNIST digit, a state at the level k=1 corresponds to a composition of parts/image patches ${\hat{x}}_{t}^{0}$ that are each manipulated by affine transform parameters $a_{t}^{0}$. A sequence of transformed patches is then summed and added to the canvas: $\sum_{t=1}^{\tau }g({\hat{x}}_{t}^{0},a_{t}^{0})$. Hypernetworks allow the model to parameterize different “what”-“where” sequence generation models at each level of the image hierarchy, while also enabling full recursion with abstraction: the same hypernetworks can be used at each level to enable the possibility of generating an identical sequence at different spatial scales. For example, a circle “program” can be shared between scales to create an eye at a lower level and a face at a higher level with appropriate parameterizations.

Note that both the state $z^{k}$ and action $a^{k}$ are vectors: $z^{k}$ is a vector that, when passed into the $H_{\mathrm{state}}$ and $H_{policy}$ hypernetworks, generates a “program” that produces a sequence of lower-level states. For our results, we trained the model so that, for 0≤k<2, an image patch decoder generated by $H_{\mathrm{state}}$ decodes $z^{k}$ into an appropriate image patch. The dimensionality of z varied based on the task, with |z|=8,16 for the MNIST and Fashion-MNIST datasets, |z|=32 for the ETH-80 dataset, and |z|=64 for the Omniglot dataset. $a^{k}\in [-1,1]$ is a 6D vector that explicitly encodes the affine transforms (scaling, offset, rotation, and shear) used to appropriately position an image patch on a larger canvas.

We first demonstrate how our RNPs can learn to recursively parse images of handwritten digits from the MNIST dataset (34), characters from the Omniglot dataset (1), and objects from the Fashion-MNIST and ETH-80 datasets (35, 36); in each of these cases, RNPs parse input images not only into parts and subparts but also their transformations within their respective reference frames. We then characterize the embedding space of the part- and subpart-generating state vectors at two hierarchical levels of abstractions and show how learned representations at various tree depths can be composed to generate previously unseen objects.

Finally, we demonstrate the expressive power of our model by generating new images with a “grammar” based on recursive transformations (through $f_{\mathrm{policy}}$) of image primitives generated by $f_{\mathrm{state}}$.

## Results

### Image parsing into parts and subparts

We trained three-level RNPs to reconstruct MNIST digits, Omniglot characters, Fashion-MNIST objects, and ETH-80 objects. An encoder network was trained to map the input image to the top-level program (embedding vector) $z^{2}$. This encoder consisted of four convolutional layers, followed by four residual layers (37), and three linear layers with ELU activations. The final layer was split into two heads, one for generating the mean and the other for log variance of $z^{2}$. As described above, $z^{2}$ parameterizes two neural networks, $f_{\mathrm{state}}^{1}$ and $f_{\mathrm{policy}}^{1}$, via the hypernetworks $H_{\mathrm{state}}$ and $H_{\mathrm{policy}}$ respectively. The network $f_{\mathrm{state}}^{1}$ produces latent vectors $z^{1}$, corresponding to the parts (decoded as larger patches, 6×6 to 12×12 pixels). Each $z^{1}$ is then passed through the *same hypernetworks* to recursively generate networks $f_{\mathrm{state}}^{0}$ and $f_{\mathrm{policy}}^{0}$ which synthesize the subparts as latent vectors at k=0. These vectors at k=0 are not fed back into the hypernet, thereby ending the recursion, and are instead passed through a decoder to generate image patches (2×2 to 4×4 pixels). The RNP learns a part-wise representation since each part or subpart is constrained to be smaller than its parent, therefore requiring a sequence of steps to reconstruct it. Figure 2A shows examples of MNIST digits (top left), Omniglot characters (top right), Fashion-MNIST objects (bottom left), and ETH-80 objects (bottom right) generated by trained RNPs given an input image, with reconstructions at the level of parts (untiled-) and subparts (tiled images).

### Clustering of neural program space

Previous approaches to compositional representations have relied on powerful formalisms such as probabilistic programs (1) but a notable challenge has been the absence of a continuous program space that can be interpretably manipulated and optimized. RNPs address this challenge by using hypernetworks to generate neural programs from vector representations. Since RNPs use the same hypernetworks to generate programs at all levels, we investigated whether programs at different tree depths inhabit different regions of |z|-dimensional space. Specifically, do programs representing digits cluster separately from programs representing parts? Analyzing the embedding space of $z^{2}$ and $z^{1}$ vectors for MNIST digits and Omniglot characters, we found that the $z^{2}$ and $z^{1}$ “neural program” vectors do cluster separately (Fig. 2B and C).

To test whether this “neural program” embedding space is smooth enough to allow interpolation for novel programs to be generated, we investigated the regions between learned $z^{2}$ and $z^{1}$ program clusters. Specifically, we used linear interpolation between the centers of two clusters to sample new program vectors. These vectors, when passed through the trained hypernetworks, produced programs that generated novel images (Fig. 2D), showing that the model can exploit the latent structure of the program embedding space to synthesize previously unseen patterns by combining the learned parts.

### Compositionality and transfer learning

**Fig. 3.**
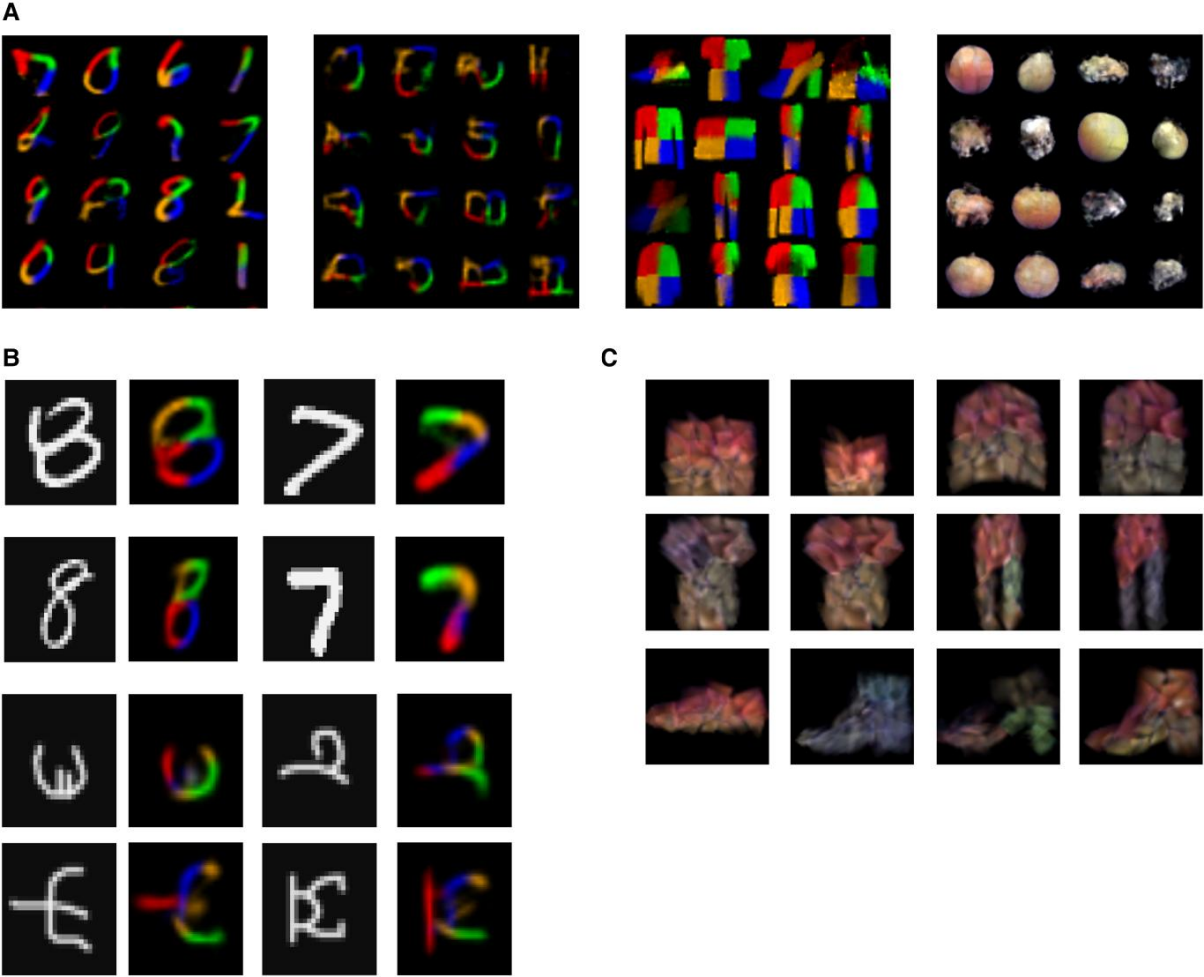
Compositionality and transfer learning. A) Sampling $z^{2}$ from the prior distribution N(0,I) for models trained on MNIST (first-), Omniglot (second-), Fashion-MNIST (third-), and ETH-80 (fourth column) datasets. Part order as in Fig. 1. B) RNPs trained on a training subset of MNIST digit classes (top; see text) and Omniglot character classes (bottom) are able to explain novel examples from unseen classes and synthesize their parts. C) Example Fashion-MNIST objects generated by an RNP with its hypernetwork $H_{\mathrm{state}}$ trained on the ETH-80 dataset.

Compositionality is the ability to compose a large (possibly infinite) number of objects using a finite set of compositional elements. The RNP model was designed with compositionality as an important goal. We have already demonstrated how the model can sample program space in regions outside those representing the trained data to generate new objects and compositional elements by interpolating between clusters of $z^{2}$ and $z^{1}$ vectors (Fig. 2D). Additionally, by sampling $z^{2}∼N(0,I)$ (the prior distribution assumed in the RNP model for $z^{2}$), the model can generate novel characters and objects by synthesizing learned primitives in different, often novel, combinations of parts (Fig. 3A).

We also tested the compositional ability of our model in two transfer learning tasks. Firstly, we trained RNPs on all MNIST classes but one (e.g. 7 or 8), and on the Omniglot training dataset (1) designed to test transfer learning. By adapting only the weights of the encoder network (but not the decoder hypernetworks $H_{\mathrm{state}}$ and $H_{\mathrm{policy}}$), RNPs were able to synthesize parts for unseen classes (Fig. 3B).

To further explore generative transfer in the model, we adapted an RNP trained on the ETH-80 dataset to generate colored Fashion-MNIST objects by keeping the $H_{\mathrm{state}}$ network the same and training new encoder and $H_{\mathrm{policy}}$ networks. The model’s ability to achieve this task is illustrated in Fig. 3C.

**Fig. 4.**
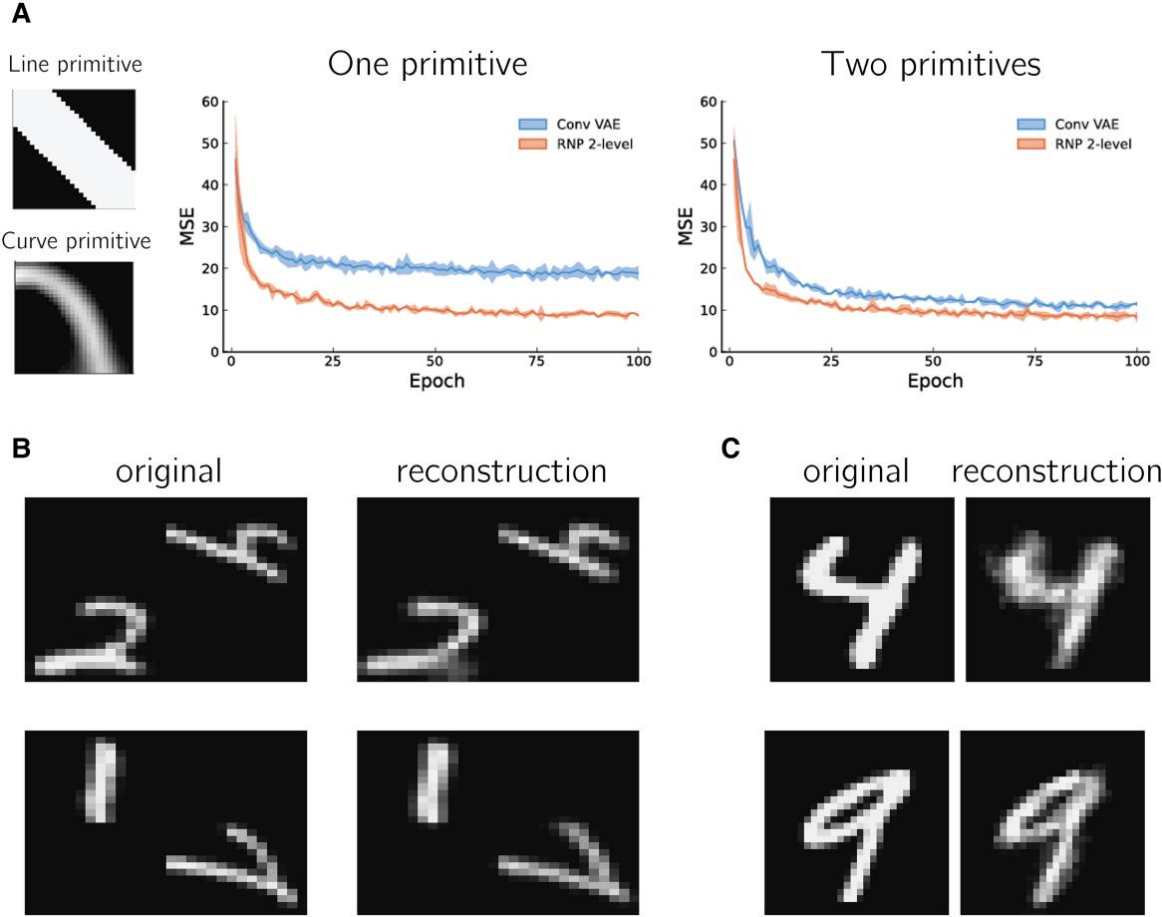
Recursion and transformations enable efficient reuse of primitives. A) RNPs were trained to reconstruct MNIST digits using either a single line primitive (left, top) or the line primitive and a curve primitive (left, bottom). RNPs with one or two primitives outperform convolutional VAEs with a similar number of parameters when both models are constrained to use the provided primitive(s), as demonstrated by the plots on the right showing the MSE reconstruction loss on the test set for each training epoch. Solid lines indicate mean, ribbons indicate standard deviation (three models per group). Epochs correspond to training on the full MNIST training set (50,000 images). B) A four-level RNP can generate digit pairs at different locations and transformations in a larger image. Left columns: original pair; right columns: RNP model reconstruction. C) MNIST digits generated by the three-level truncation of the learned four-level model in (B) using the output of an encoder trained on centered (non-transformed) MNIST digit input images (see text for details).

We next investigated whether the hierarchical, recursive compositionality used by RNPs confers on them any advantages over traditional noncompositional generative models such as a variational autoencoder (VAE). We trained an RNP with only one or two primitives—a straight line, or a straight line and a curve—and compared the generative performance of such an RNP to a standard convolutional VAE with a similar number of parameters as the RNP and using the same primitive(s) as filters in the final deconvolution layer. We found that RNPs outperform VAEs in terms of mean squared error (MSE) reconstruction loss on MNIST datasets (Fig. 4A).

Finally, to illustrate the recursive ability of the model, we trained a four-level RNP (recursion depth=3) on larger images containing pairs of MNIST digits at different locations (Fig. 4B, left panels). We found that the RNP model was able to successfully learn to encode and generate these larger images (Fig. 4B, right panels). Furthermore, examining the learned four-level RNP revealed that it had learned a three-level RNP on individual MNIST digits—it was sufficient to train an encoder network on centered MNIST digits to utilize this three-level RNP (taken from the trained four-level model) to generate individual MNIST digits without any additional training (Fig. 4C).

## Discussion

This article introduces RNPs, a new model for learning hierarchical and tree-structured representations. The model uses hypernetworks, neural networks that generate other neural networks, to learn state-action sequences at multiple levels of abstraction, allowing flexible composition of learned primitives and implementing a recursive “grammar.” We demonstrated the model’s ability to explain objects in images using a hierarchy of learned parts and their transformations. Beyond images, we expect the model to be useful for learning hierarchical solutions to problems in other domains as well, such as audio and video analysis, and modeling sensory-motor behavior.

The general architecture of RNPs (Fig. 1) is consistent with recent models of the neocortex that emphasize sensory-motor representations across cortical areas (27, 31, 38, 39). Indeed, the recursive, language-like information-processing architecture used by RNPs may suggest new ways of modeling how the human brain could learn and represent concepts by composing sensory-motor primitives into a dynamic parsing tree. Composing continuous primitives like lines, curves, faces, etc. (which in this scheme are also recursively defined) with representations of sensory-motor programs can also account for the flexibility and fluidity of the concepts humans can construct.

We note that the RNP architecture is strongly related to deep active inference (40, 41), which infers actions necessary to minimize prediction errors (6, 42, 43). In RNPs, the hypernetwork $H_{\mathrm{policy}}$ plays the same role, though it generates functions (policies) instead of individual actions. Inferring policies provides a type of hierarchical generalization over inferring actions. Furthermore, our model operates in a continuous state-space, allowing interpolation and generalization, whereas most implementations of active inference have been discrete.

The results presented in this article suggest several potential directions for future research. Hypernetworks describing different data modalities (e.g. audio and visual) could be combined to generate richer multi-modal neural programs. Alternate neural implementations of RNPs, using, for example, an embedding approach (44, 45) or gain modulation (46, 47) instead of hypernetworks, are also worthy of investigation, given their neurobiological plausibility (27). In the work presented here, RNPs were trained on simple image datasets containing images of single objects without background clutter. From such images, the model was able to learn parts of objects and the relationship between parts. Parsing more complex images containing multiple objects and background clutter would require changes to the model that allow for multiple objects with different statistics, as well as figure-ground segregation. We are exploring incorporating such capabilities into the RNP model to enable part-whole learning for complex image datasets.

For our results, we used end-to-end backpropagation to train our multi-level RNP networks. However, following predictive coding (48), gradient descent can also be performed using local prediction errors (49–51). We intend to explore this more biologically plausible implementation of learning in RNPs in future work.

In this article, we used a deep encoder network that translated an image into the mean and log variance of a top-level state vector $z^{2}$ that hierarchically reconstructs the image in a single top-down pass through the generative part of the RNP. An alternative encoding strategy could be to use Active Predictive Coding (APC) (25, 26), which intelligently and hierarchically samples the input image and uses prediction errors to infer an appropriate encoding vector. A related method in line with the predictive coding model (48) is to use an iterative gradient-based procedure, starting from a zero or random $z^{2}$ vector, computing prediction errors made by the generative model, and integrating them to eventually arrive at an estimate for $z^{2}$ that best reconstructs the input image. We are currently exploring these prediction error-driven approaches.

Also worth investigating are predictive coding-based alternatives to the variational approach to RNPs used in this article; in a predictive coding implementation of RNPs, predictions from parent nodes would be conveyed to child nodes and prediction errors from child nodes to parent nodes would be used to continually update state and action estimates at all levels (25, 26, 28, 29, 48). Finally, the RNP loss function could be modified to include rewards and costs, potentially opening the door to modeling difficulties in concept learning and planning observed in psychiatric disorders such as anxiety and depression.

## Materials and methods

**Fig. 5.**
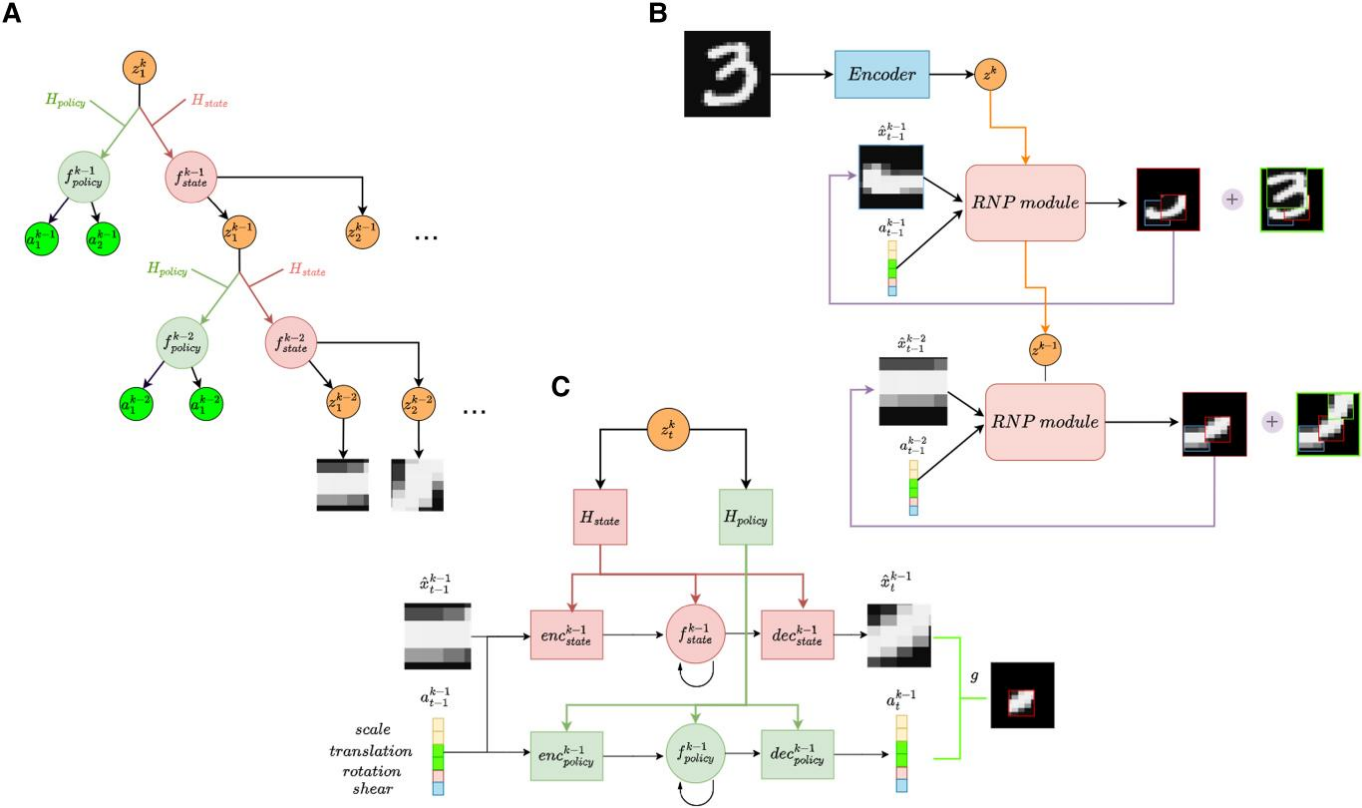
RNP model implementation. A) Schematic representation of a recursive neural program using state-action (“sensory-motor”) networks at multiple levels (identical to Fig. 1C, right). B) The generative process for an RNP. Images are encoded into $z^{k}$ program vectors using an encoder network. $z^{k}$ generates the parameters for an RNP module (see C), which receives the previous predicted image patch ${\hat{x}}_{t-1}^{k-1}$ and previous affine transform parameters $a_{t-1}^{k-1}$ to generate a sequence of $z^{k-1}$ program vectors representing parts and their transformations. C) An RNP module consists of two neural networks (“hypernetworks”) $H_{state}$ and $H_{policy}$ that each generate the parameters for the following networks as shown: (1) an encoder network that concatenates the previous generated image ${\hat{x}}_{t-1}^{k-1}$ and affine transform parameters $a_{t-1}^{k-1}$ and feeds them into (2) the $f_{\mathrm{state}}$ and $f_{\mathrm{policy}}$ recurrent neural networks (RNNs); and (3) decoder networks that decode the hidden state of the $f_{\mathrm{state}}$ and $f_{\mathrm{policy}}$ RNNs into an image patch and affine transform parameters, respectively.

A program at tree depth k is represented by a “state” vector $z_{t}^{k}$, which generates (via neural networks—see below) a fixed-length sequence of $\tau^{k-1}$ lower level states $S^{k-1}=[z_{1}^{k-1},\ldots ,z_{\tau^{k-1}}^{k-1}]$ and corresponding actions $T^{k-1}=[a_{1}^{k-1},\ldots ,a_{\tau^{k-1}}^{k-1}]$ (in the current paper, the states are parts and actions are transformations of the parts). Each lower level state $z_{t}^{k-1}$ can be decoded into an image patch $x_{t}^{k-1}$ that corresponds to a part (e.g. stroke or other image feature), which is then transformed according to $g(x_{t}^{k-1},a_{t}^{k-1})$ to place it on a “canvas” (here a refers to parameters of an affine transform on a grid and g is the bilinear interpolation function (52)). The transformed images are added together on the canvas at each time step to generate the target image represented by higher-level state $z^{k}$ (Fig. 5B). This method allows the model to reuse the same parts with different transformations. For example, if $z_{t}^{k}$ represents an image of the digit “4”, $S^{k-1}$ can represent three straight lines, and $T^{k-1}$ represents the transformations that orient and place them in the configuration of a “4” (Fig. 1A).

The above model can be made recursive, with generation performed in a depth-first manner (Algorithm 1): each $z_{t}^{k-1}$ above generates lower level states $S^{k-2}=[z_{1}^{k-2},\ldots ,z_{\tau^{k-2}}^{k-2}]$ and actions $T^{k-2}=[a_{1}^{k-2},\ldots ,a_{\tau^{k-2}}^{k-2}]$, and so on for each level until the lowest level. The next state $z_{t+1}^{k-1}$ begins after the current state $z_{t}^{k-1}$ terminates.

In a three-level RNP (Fig. 5A,B), the top-level program $z^{2}$ parameterizes two recurrent neural networks (RNNs) $f_{\mathrm{state}}^{1}$ and $f_{\mathrm{policy}}^{1}$ via hypernetworks (state hypernetwork $H_{\mathrm{state}}$ and policy hypernetwork $H_{\mathrm{policy}}$) (24). As shown in Fig. 5C, each hypernetwork generates the following set of parameters for the neural networks at the lower level: single-layer encoders $enc_{\mathrm{state}/\mathrm{policy}}^{1}$ which compute ${\hat{e}}_{\mathrm{state}/\mathrm{policy}}^{1}=enc_{\mathrm{state}/\mathrm{policy}}^{1}({\hat{x}}_{t}^{1},a_{t}^{1})$ and feed into the $f_{\mathrm{state}/\mathrm{policy}}^{1}$ networks respectively; $f_{\mathrm{state}/\mathrm{policy}}^{1}$, RNNs with hidden state of size |z|; and decoders $dec_{\mathrm{state}/\mathrm{policy}}^{1}$ that generate respectively an image patch ${\hat{x}}_{t+1}^{1}$ for the state network and affine transform parameters $a_{t+1}^{1}$ for the policy network (scaling, translation, rotation, and shear for each patch). The hypernetworks also provide initialization values ${\hat{x}}_{0}^{1},\;a_{0}^{1}$ to initialize the sequence generation.

We train the model described above by exploiting the end-to-end differentiability of the architecture, minimizing the reconstruction loss between the canvas containing the sum of all transformed parts and the input image x.


$$L={\left\|\sum_{t_{2}=1}^{\tau^{2}}g\left(\sum_{t_{1}=1}^{\tau^{1}}g({\hat{x}}_{t_{1}}^{1},a_{t_{1}}^{1}),a_{t_{2}}^{2}\right)-x\right\|}_{2}^{2}$$ (1)

where $\tau^{2}$ and $\tau^{1}$ are the number of level-2 and level-1 time steps, respectively. We note that RNPs can be trained one depth at a time to decrease training time and computational resources. In order to facilitate a more interpretable program space, we can regularize Eq. 1 by adding the reconstruction error at the level of parts to the loss:


$$\frac{1}{\tau^{2}}{\left\|\sum_{t_{2}=1}^{\tau^{2}}g({\hat{x}}_{t_{2}}^{2},a_{t_{2}}^{2})-x_{t_{2}}^{patch}\right\|}_{2}^{2}$$ (2)

where $x_{t_{2}}^{\mathrm{patch}}$ is the image patch generated by performing bilinear interpolation on x with the inverse of the affine transform parameters $a_{t_{2}}^{2}$. This is equivalent to zooming into the image at those coordinates, as opposed to scaling down.

To allow probabilistic sampling of programs, we can express an RNP as a structured form of variational autoencoder (VAE) (7) to learn an approximate posterior $q(z^{K}|x)\approx p(z^{K}|x)$ of an image x given prior $p(z^{K})∼N(0,I)$, where $z^{K}$ is the highest level state vector and I is the identity matrix. Following the standard approach for VAEs, we use an encoder network to parameterize the approximate posterior $q(z^{K}|x)$ and regularize Eq. 1 by adding the Kullback–Leibler term KL(q‖p) (7).

Algorithm 1 summarizes the inference process for a recursive neural program given an input image. The results of this inference are used for training the model as described above (training steps are not shown in Algorithm 1).

## Data Availability

All code is publicly available at https://github.com/FishAres/RNP6
